# The impact of oat structure and β-glucan on *in vitro* lipid digestion

**DOI:** 10.1016/j.jff.2017.09.011

**Published:** 2017-11

**Authors:** Myriam M.L. Grundy, Janina Quint, Anne Rieder, Simon Ballance, Cécile A. Dreiss, Kathryn L. Cross, Robert Gray, Balazs H. Bajka, Peter J. Butterworth, Peter R. Ellis, Peter J. Wilde

**Affiliations:** a Biopolymers Group, Diabetes and Nutritional Sciences Division, King’s College London, Franklin-Wilkins Building, 150 Stamford Street, London SE1 9NH, UK; b Quadram Institute Bioscience, Norwich Research Park, Colney, Norwich NR4 7UA, UK; c University of Vienna, Department of Nutritional Sciences, Althanstraße 14 (UZA II), 1090 Vienna, Austria; d Nofima, Norwegian Institute for Food, Fisheries and Aquaculture Research, PB 210, N-1431 Ås, Norway; e Institute of Pharmaceutical Science, King’s College London, Franklin-Wilkins Building, 150 Stamford Street, London SE1 9NH, UK

**Keywords:** BG1, oat β-glucan of high MW, BG2, oat β-glucan of medium MW, BG32, oat bran rich in β-glucan, FFA, free fatty acids, MW, molecular weight, WPI, whey protein isolates, Oat β-glucan, Lipolysis, *In vitro* digestion, Viscosity, Oat structure, Molecular weight

## Abstract

Oat β-glucan has been shown to play a positive role in influencing lipid and cholesterol metabolism. However, the mechanisms behind these beneficial effects are not fully understood.

The purpose of the current work was to investigate some of the possible mechanisms behind the cholesterol lowering effect of oat β-glucan, and how processing of oat modulates lipolysis. β-Glucan release, and the rate and extent of lipolysis measured in the presence of different sources of oat β-glucan, were investigated during gastrointestinal digestion.

Only a fraction of the original β-glucan content was released during digestion. Oat flakes and flour appeared to have a more significant effect on lipolysis than purified β-glucan.

These findings show that the positive action of β-glucan is likely to involve complex processes and interactions with the food matrix. This work also highlights the importance of considering the structure and physicochemical properties of foods, and not just the nutrient content.

## Highlights

• The structure of oat tissue is an important factor for determining its influence on (*in vitro*) lipid digestion.
• β-glucan release from oat cell walls during digestion was not complete.
• Processing of oats affects the rate and extent of lipolysis.
• Viscosity is not the only factor affecting lipolysis.

## Graphical abstract

The effect of oat β-glucan on lipid digestion relies on the delivery form of the polymer.

**Figure:**
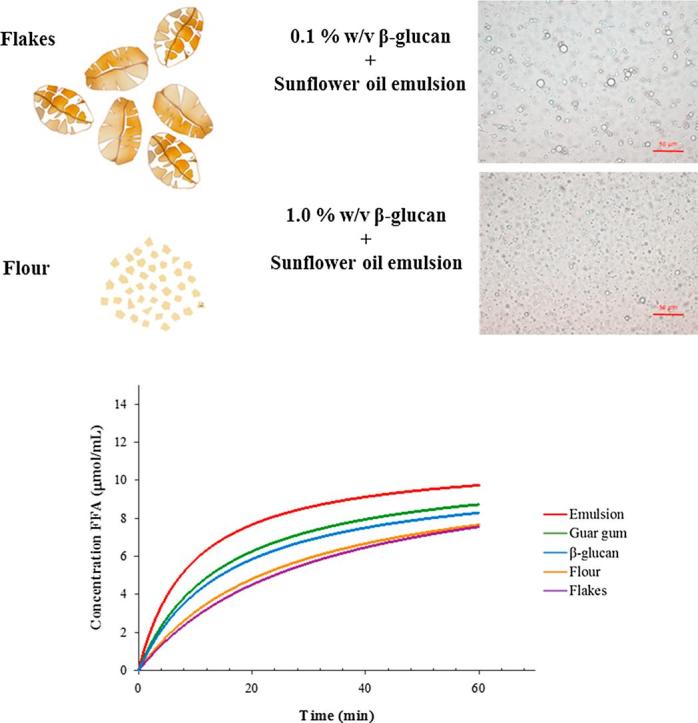

## 1 Introduction

The oat grain (*Avena sativa* L.) is a complex food matrix made up of the groat (kernel) surrounded by an outer layer known as the hull (Miller & Fulcher, 2011). The hull is removed from the groat before further processing (Decker, Rose, & Stewart, 2014). Oat is commonly consumed whole as oat flakes (i.e. rolled oat), and is composed of three distinct anatomical parts: the pericarp and testa (bran), the endosperm, and the embryo (germ). The bran constitutes a coarse, fibrous coat that is difficult to separate from the endosperm, and contains the aleurone and subaleurone layers that are rich in proteins, lipids, minerals, vitamins, and cell wall polysaccharides such as mixed-linkage β-glucan, a form of water-soluble dietary fibre (Miller and Fulcher, 2011, Yiu, 1986). The endosperm is rich in starch while the germ is an important source of proteins and lipids. Therefore, the structure and composition of the aleurone, subaleurone and endosperm cells differ from each other. Aleurone and subaleurone cells are somewhat cuboidal with a thick cell wall whereas the endosperm is composed of elongated cells possessing relatively thin cell walls.

Consumption of foods containing cereal β-glucan have been shown to lower blood total and LDL-cholesterol concentrations and this effect is related to a reduced risk of coronary heart disease (Othman et al., 2011, Theuwissen and Mensink, 2008). The evidence for these beneficial effects of β-glucan in oats has been reviewed and accepted by the Food and Drug Administration (FDA) (US Food & Drug Administration, 1997) and, later by the European Food Safety Authority (EFSA) (EFSA NDA Panel, 2009), as a valid health claim. However, the mechanisms involved in the lowering of blood cholesterol remain unclear. The observed positive effects on blood cholesterol levels are thought to be mainly related to the physicochemical properties of the oat β-glucan on a molecular scale, specifically its weight-average molecular weight (MW), solubility and the subsequent viscosity it generates (Wolever et al., 2010).

The most documented mechanism seems to be the ability of β-glucan to interfere with the recycling of bile salts and the impact on cholesterol metabolism (Gunness et al., 2016, Gunness and Gidley, 2010, Gunness et al., 2016). However, the physiology of cholesterol metabolism is complex and is likely to be also related to lipid metabolism (Carey & Hernell, 1992). Since cholesterol and lipids are both hydrophobic, their digestion and absorption are interconnected. For instance, cholesterol and the products of lipolysis are incorporated together into bile salts mixed micelles before absorption by the enterocytes. The proportion of cholesterol present in the micelles is influenced by the lipid composition (Vinarov et al., 2012). However, this interconnectivity is not always observed *in vivo*, certainly not when investigating the effect of water-soluble forms of dietary fibre, such as β-glucan, on blood triacylglycerol and cholesterol concentrations (Brown et al., 1999, Othman et al., 2011, Whitehead et al., 2014). There is little detail available about the processes by which β-glucan may directly affect lipid digestion (Lairon, Play, & Jourdheuil-Rahmani, 2007). However, evidence suggests that water-soluble fibres may interfere with lipid emulsification (Pasquier et al., 1996, Zhai et al., 2016) or lipase activity (Wilcox, Brownlee, Richardson, Dettmar, & Pearson, 2014), and thus alter bile salt and cholesterol homeostasis.

While the effects of processing on the physicochemical properties of individual oat constituents, notably β-glucan, have been investigated and shown to influence the extent of their bioactivity (Tosh and Chu, 2015, Tosh et al., 2010, Yiu, 1986, Yiu et al., 1987), the impact of processing of oat groats, including cooking, on lipid digestion and absorption has received little attention. Moreover, processing may also affect the structural characteristics of the cereal matrix on a macroscale, which can have an impact during digestion. This is an aspect that so far has not been studied in detail. Further investigation of the effect of the structural integrity of the oat matrix on lipolysis was required to reach a better understanding of the conflicting *in vivo* results in the literature obtained with different oat products.

Therefore, the aim of the present work was to investigate the role played by β-glucan containing foods on *in vitro* lipid digestion and so gain a better understanding of how oats, and the way they are prepared and consumed may impact on blood lipid and cholesterol concentrations. To address these objectives, we monitored β-glucan release and macronutrient digestibility from complex oat matrices, and observed, by scanning electron microscopy (SEM), the oat particles recovered after digestion. Then, we determined the effect on lipolysis of β-glucan from different sources, varying in complexity, composition and type of processing. Finally, we assessed the possible interaction between lipids and purified polymers (guar galactomannan, a positive control, and β-glucans) using confocal microscopy.

## 2 Materials and methods

**Fig. 1:**
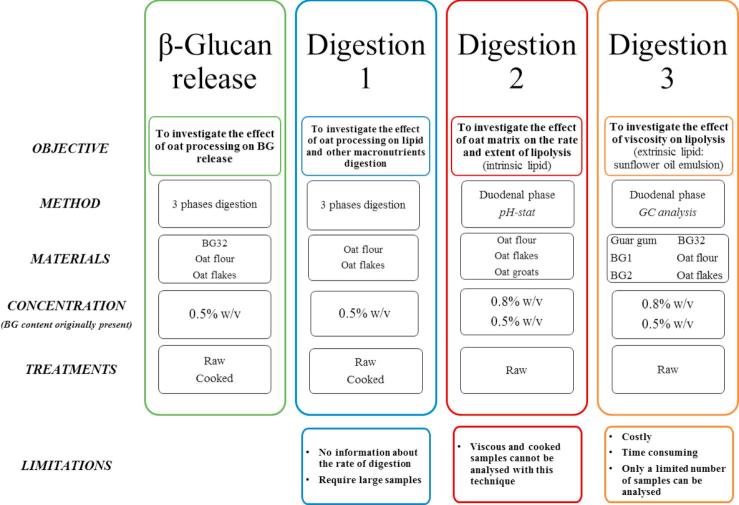
Summary of the methodology of the *in vitro* digestions applied to oat materials and guar gum to follow β-glucan (BG) release and its effect on lipolysis.

In the present work, three complementary *in vitro* digestion methods were employed to overcome the limitations associated with each technique and the high viscosity of some of the samples. A summary of the experiments performed can be found in Fig. 1. The experiments were designed to assess how oat materials behaved following different forms of processing, by investigating their degradation during digestion (Digestion 1), β-glucan release, and the subsequent effect on lipid digestion (both intrinsic and extrinsic, Digestions 2 and 3). Digestion 3 also provided a comparison of processed oats with the effect of β-glucan alone on lipolysis.

### 2.1 Materials

Oat groats and flakes from the Belinda variety were obtained from Lantmännen Cerealia, Moss, Norway. Oat flour was produced from the Belinda oats by milling the flakes on a laboratory hammer mill (Retsch, Model ZM100, Retsch GmbH, Haan, Germany) with a 0.5 mm mesh. Extracted oat β-glucan of high MW (BG1, weight-average MW 730 × 10^3^ g/mol, purity ∼91% dry-weight basis, d.b.) was a generous gift from Dr. Susan Tosh at Agricultural and Agri-Food Canada. Swedish Oat Fiber (Swedish Oat Fiber AB, Bua, Sweden) provided oat bran rich in β-glucan (BG32) and medium MW β-glucan (BG2, brand name BG90, weight-average MW 470 × 10^3^ g/mol, purity ∼91% d.b.). Guar gum flour (Meyprogat M150; galactomannan weight-average MW 2500 × 10^3^ g/mol, purity ∼88% d.b.) was generously provided by Dr. Graham Sworn (Danisco, Paris, France). The methods used for the determination of polysaccharide MW are included in the next section.

α-Amylase from human saliva (1000 U/mg of solid), pepsin from porcine gastric mucosa (2884 U/mg of solid), bovine bile extract, pancreatin (40 U/mg of solid based on lipase activity), and sunflower oil were purchased from Sigma (Poole, Dorset, UK). Powdered whey protein isolate (WPI) was donated by Davisco Foods International (Le Sueur, MN, USA). Internal standards for gas chromatography (GC) analysis, C15:0 (pentadecanoic acid, monopentadecanoin, 1,3-dipentadecanoin, and tripentadecanoin), were purchased from Nu-Chek-Prep, Inc (Elysian, MN, USA). Sodium dihydrogen phosphate (99%), disodium hydrogen phosphate (99%), sodium chloride (99.8%), calcium chloride (99%), potassium chloride (99%), potassium dihydrogen phosphate (99%), sodium bicarbonate (99.5%), magnesium chloride hexahydrate (99%), and ammonium carbonate (99.5%) were purchased from Sigma-Aldrich Chemical Co. (Poole, UK).

### 2.2 Physical and chemical characterisation of materials

A full description of the proximate and polysaccharide weight-average MW (calcofluor and SEC-MALLS methods) analyses of the oat groats, flakes and flour, BG32, BG1, BG2, and guar gum at baseline can be found elsewhere (Grundy et al., 2017). The β-glucan content of the original oat samples was determined using the AOAC method (AOAC 995.16). In the β-glucan release experiments described in Section 2.3, the oat samples underwent *in vitro* digestion (with or without digestive enzymes), and the β-glucan content in the aqueous phase was determined. To do this, the digested samples were first centrifuged (1800*g* for 10 min), the polymer contained in the supernatant was precipitated in ethanol and then analysed using an enzymic method based on a cereal mixed-linkage β-glucan kit from Megazyme (Bray, Wicklow, Ireland). The released β-glucan (i.e., solubilised fraction) was expressed as a percentage of the total β-glucan originally present in the sample. The weight-average MW of the released β-glucan was determined using the calcofluor method (Grundy et al., 2017). Each measurement was carried out in duplicate.

Digested samples (Digestion 1) were analysed by Eurofins Agroscience Services Ltd (Crimplesham, UK) using standardised methods for protein (Dumas, N x 6.5), lipid (BS 4401 pt4:1970), starch (Enzymic method, ISO 15914), ash (combustion at 525 °C) and moisture (oven-dried at 102 °C). Each measurement was performed in duplicate.

### 2.3 In vitro digestion and quantification of β-glucan release

#### 2.3.1 Standardised in vitro digestion

Two sets of experiments used the three steps (oral, gastric and duodenal) standardised *in vitro* digestion protocol recommended by the Infogest COST Action (Minekus et al., 2014). The first series of experiments were designed to measure the release of β-glucan overtime into the incubation mixture during the digestion of raw and cooked BG32, oat flour and oat flakes. Due to the heterogeneity of the sample, the standardised protocol was scaled down by a factor of 8 (final volume of 5 mL, containing either 2.08 g of flakes/flour or 0.285 g of BG32) in order to analyse the entire volume thereby preventing sampling error. β-Glucan release from the oat samples during the oral, gastric and duodenal stages was determined as described in Section 2.2. The second series of experiments (Digestion 1) were scaled up (final volume of 1020 mL, containing 100 g of flakes or flour) to measure the macronutrient composition (i.e., starch, protein and lipid) remaining in the raw oat flour and flakes following digestion (Section 2.2 for methods).

The raw oat materials were added to simulated salivary fluid so that the β-glucan concentration in the oral phase was equal to 0.5% (w/v). After 2 min of incubation at 37 °C pH 7, the samples went through gastric (120 min at pH 3) and duodenal (120 min at pH 7) digestions. The hydrothermally processed (cooked) samples for the β-glucan release experiments were obtained by adding deionised water (84% of total weight of starting material) to the raw oats, and then placing these samples into a boiling water bath. After 10 min of cooking, each sample was digested following the same protocol as that used for the raw oats. Each set of experiments was performed in duplicate.

#### 2.3.2 Duodenal digestion with the pH-stat (Digestion 2)

The *in vitro* pH-stat duodenal digestion assay was adapted from a previous protocol (Grundy, Wilde, Butterworth, Gray, & Ellis, 2015). The reaction system had a final volume of 37.5 mL and contained oat materials dissolved in 1 wt% WPI solution, 12.5 mM bile salt, 150 mM NaCl, 10 mM CaCl_2_, and either 17 mg/mL pancreatin (digestion) or 10 mM phosphate buffer (blank). The quantities of the oat materials used corresponded to β-glucan concentrations of either 0.5 or 0.8 wt%. In these experiments and Digestion 3 experiments, we used 0.8% β-glucan was used, in addition to 0.5%, to assess the effect of increased starting β-glucan concentration, and thereby viscosity, on lipolysis. The rate and extent of free fatty acids (FFA) released during lipolysis of the intrinsic oat lipids were monitored by titration with 0.10 M NaOH for 60 min at 37 °C, pH 7. Calculations of FFA release and further details of the method can be found elsewhere (Grundy et al., 2015). All lipolysis experiments were carried out in triplicate.

#### 2.3.3 Duodenal digestion combined with GC analysis (Digestion 3)

To investigate the effect of polymer concentration and MW, dispersions comprising 1.0 and 1.6% (w/w) of guar gum or β-glucan were obtained by slowly sprinkling the polymer powder into a rapidly swirling vortex of 10 mM phosphate buffer, pH 7. The mixture was heated at 80 °C for 2 h and then left at room temperature overnight. Emulsions were obtained by pre-emulsifying 3.2 or 6.4 wt% oil in 1% (w/w) WPI solution using an homogeniser (Ultra-Turrax T25, IKA® Werke, from Fisher Scientific Ltd.) for 1 min at 11,000 rpm. The pre-emulsion was then sonicated with an ultrasonic processor (Sonics & Materials Inc, Newtown, USA) at 70% amplitude for 2 min. The average droplet size (d_32_) of the emulsions was 2.0 µm as measured by laser diffraction (LS13320^®^, Beckman Coulter Ltd., High Wycombe, UK).

The emulsion containing the polymer was prepared by adding 21.28 mL of 6.4 wt% emulsion into 40 mL of polymer solution, and stirring the mixture for 30 min; 3.83 mL of this emulsion/polymer solution was used for the digestion. For the more complex sources of β-glucan (i.e., flakes, flour and BG32), 3.83 mL of 3.2 wt% emulsion was added directly to the oat materials to obtain a final concentration of either 0.5 or 0.8% β-glucan. In a 50 mL tube, 0.8 mL of bile salt solution, 0.135 mL of NaCl and 0.135 mL of CaCl_2_ were added to the polymer or oat material mixture. The system was adjusted to pH 7 and then 0.1 mL of freshly prepared pancreatin solution (digestion) or 10 mM phosphate buffer (blank) was added. The final volume of the reaction system was 5 mL and its composition was 0.8 and 0.5% β-glucan (from either the pure polymer or oat materials), 0.8 wt% lipid, 10 mM bile salt, 150 mM NaCl, 10 mM CaCl_2_ plus either 17 mg/mL pancreatin (digestion) or 10 mM phosphate buffer (blank). The tubes were then rotated in an incubator set at 37 °C. A separate digestion experiment was performed for each reaction with sampling times of: 0, 2, 5, 10, 15, 30 and 60 min following initiation of the reaction. Control samples, containing the oat materials without the emulsion, were analysed to account for the lipolysis of the oat lipids. Each digestion reaction was repeated two times.

The lipids present in the aqueous phase of the samples were extracted at different time points using a 2:1 chloroform – methanol (v/v) solution containing C15:0 internal standards (Folch, Lees, & Sloane Stanley, 1957). The chloroform – methanol (v/v) solution was added directly to the tubes. The mixture was centrifuged at 2500*g* for 10 min at 4 °C and then 1.5 mL of the chloroform layer was collected and transferred into a vial. The samples were evaporated to dryness in a heated centrifugal evaporator (Genevac SF50, Genevac Ltd., Ipswich, UK) for approximately 60 min. MSTFA (N-Methyl-N-(trimethylsilyl) trifluoroacetamide) derivatives were obtained through transesterification by dissolving the dried samples in 0.1 mL of MSTFA and then leaving at room temperature for 5–10 min. To each vial, 0.1 mL of heptane was added. The vials were hermetically sealed and left overnight at room temperature. MSTFA derivatives were separated and quantified using gas chromatography (7890A, Agilent Technologies UK Ltd, Wokingham, UK) equipped with a SGE HT8 capillary column (dimensions: 50 m length × 220 mm internal diameter × 0.25 µm film thickness) and a flame ionization detector (FID). The injection volume was 1 µL and the inlet type was a split/splitless operated in split mode with split ratio set to 20:1. The carrier gas was hydrogen set at a flow rate 1 mL/min (constant pressure mode). The oven was temperature programmed as follows to optimise the separation of the derivatives: initial temperature 250 °C for 1.5 min followed by ramp at 10 °C/min to 350 °C and then held for 60 min. The total runtime was 71.5 min. The detection was performed with the FID set at 400 °C. Finally, the fatty acids were identified by comparing their relative retention time with those of the standards (C15:0 species). The concentration of each species was determined using the internal standards and expressed in μmol/mL.

### 2.4 Microstructural characterisation of oat materials (SEM)

The original (baseline) oat materials (i.e., groat, flakes, flour and BG32) were attached to stubs without fixation and dehydration. The groat samples were cut in cross-section before mounting on the stubs. Groat and flakes were attached with silver paint and the two types of flour samples were attached using sticky tabs.

The raw, cooked and digested oat samples were fixed in 2.5% glutaraldehyde in 0.1 M piperazine-N,N′-bis(2-ethanesulphonic acid) (PIPES) buffer (pH 7.4). After washing with 0.1 M PIPES buffer, an aliquot of each sample was transferred onto the centre of a small square of filter paper which had been pre-folded into three equal parts in both directions and then opened out. The filter paper was then folded and inserted into a critical point drying capsule and dehydrated in a series of ethanol solutions (10, 20, 30, 40, 50, 60, 70, 80, 90, and 3 × 100%). Samples were dried in a Leica EM CPD300 Critical Point Dryer using liquid carbon dioxide as the transition fluid. The parcels were carefully unfolded in a clean plastic petri dish and the dry flour material was mounted onto SEM stubs via sticky tabs by flicking the back of the filter paper in the direction of the stub. Groat or flakes were attached to stubs using silver paint. The groat samples were cut in cross-section before mounting on the stubs.

The samples were coated with gold in an agar high resolution sputter-coater apparatus. Scanning electron microscopy was carried out using a Zeiss Supra 55 VP FEG SEM, operating at 3 kV.

### 2.5 Analysis of emulsion stability by confocal microscopy

The emulsions, either alone or in the presence of the polymers over a range of concentrations (0.1 to 1.0% (w/v) of polymer), were prepared as described in Section 2.3.3 and then visualised using a confocal laser scanning microscope (SP1 CLSM, Leica Microsystems, Mannheim, Germany). Nile red (15 µL of 1 mg/mL in dimethyl sulphoxide added to 700 µL of emulsion/polymer mixture) was used to detect the lipids and the images were captured using a 20× (N.A. 0.5) objective lens. The samples were excited using an argon laser at 488 nm and the fluorescence emitted by the samples was detected at 510–590 nm.

### 2.6 Statistical analysis

The data were analysed using SPSS version 22.0. For all tests, the significance level was set at *P* < 0.05 (2 tailed) and the data were expressed either as means of duplicates or means of triplicate ± SD. The differences between materials and treatments for the β-glucan release were assessed by one-way analysis of variance (ANOVA) followed by Honest Significant Difference (HSD) post hoc test. The GC lipolysis data were analysed by one-way analysis of variance (ANOVA) but followed by Dunnett’s post hoc test. For presentation purposes and to facilitate statistical analysis, the lipolysis data were fitted by a non-linear model as follows:

(1) $$y=\frac{\mathrm{ax}}{\left(b+x\right)}$$

The constant “b” was found to give the best estimate of variability between samples (rate of change of the curves) and so was used as the dependent variable for the statistical comparisons between the data sets.

Student’s paired *t*-test was used to evaluate differences in β-glucan release between the blank and digested samples, and between the raw and cooked oat materials. The FFA release data from the pH-stat were analysed by repeated-measure ANOVA followed by Bonferroni’s post hoc test.

## 3 Results and discussion

### 3.1 β-glucan release during digestion

**Fig. 2:**
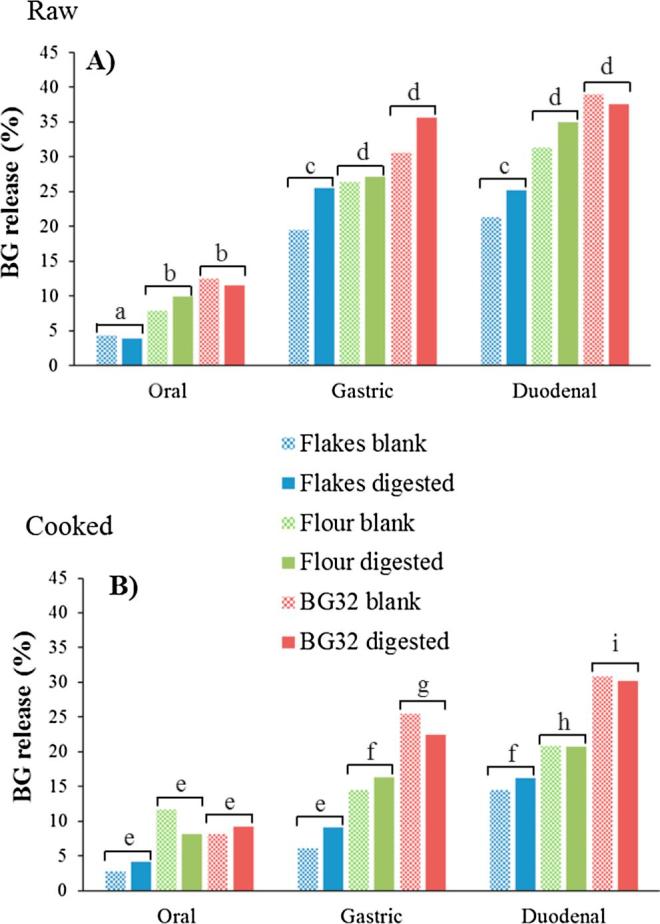
Total β-glucan released from raw and hydrothermally processed (cooked) oat flakes (blue), oat flour (green) and BG32 (red) after simulated digestion during oral, gastric and duodenal phases. The data are presented as percentages of the β-glucan originally present in the oat material (n = 2). The blank samples contain no digestive enzymes. Different letters indicate significant differences as determined by one-way ANOVA and the Honest Significant Difference (HSD) post hoc test. No statistically significant differences were found between the blank and digested samples. (For interpretation of the references to colour in this figure legend, the reader is referred to the web version of this article.)

In agreement with our previous data (Grundy et al., 2017), the amount of β-glucan solubilised from the cell walls of the oat samples was low even after 4 h of gastrointestinal digestion (Fig. 2); the maximum value was found to be ∼38% for the raw BG32. In our previous experiments, we studied dissolution kinetics of oat β-glucan in phosphate buffer only, whereas in the current investigation we determined β-glucan release during the *in vitro* oral, gastric and duodenal phases of digestion. However, digestion did not significantly increase β-glucan dissolution in any of the oat samples compared with the blank samples that contained no digestive enzymes. β-glucan release in all the oat samples increased with time, most of the solubilisation occurring during the gastric phase as previously observed by other groups (Beer et al., 1997, Rieder et al., 2017, Ulmius et al., 2011). Thus, in our experiments we found that, during the gastric phase, ∼26, 27 and 36% of β-glucan were released for raw flakes, flour and BG32, respectively, and ∼9, 16 and 23% for cooked flakes, flour and BG32, respectively. The low solubilisation of β-glucan could be explained by the stable structural organisation of the aleurone and subaleurone layers remaining in these samples that resists many forms of processing (Yiu, 1986). Also, for the flakes and flour, a large proportion of the β-glucan located in the endosperm may have resisted solubilisation even though some of the cell walls were fractured. Higher extraction yields of solubilised β-glucan could be achieved (up to 80–90%) but this necessitates harsher conditions (e.g., high extraction temperature and use of alkaline solvent) than the simulated digestion used in the present study (Lazaridou, Biliaderis, & Izydorczyk, 2007). For both raw and cooked materials, digestion of the oat flakes resulted in the lowest (P < 0.05) amount of β-glucan dissolution (duodenal values in Fig. 2: ∼25 and 16% for raw and cooked flakes, respectively) compared with the flour (∼35 and 21% for raw and cooked flour, respectively) and BG32 (∼38 and 30% for raw and cooked BG32, respectively). The differences between flour and flakes, which we reported in our recent study of dissolution behaviour in phosphate buffer only (Grundy et al., 2017), can probably be attributed to the larger particle size and thus a decreased surface area to volume ratio of the flakes. Our data are consistent with the early findings of Yiu et al. who reported that processing (i.e., cutting, rolling, and cooking) altered the structure of the cell walls which promoted β-glucan dissolution (Yiu and Mongeau, 1987, Yiu et al., 1987). However, contrary to what was shown in Yiu’s work, the hydrothermal processing conditions employed in our experiments seemed to hinder the solubilisation of the polymer (P < 0.05). This is probably because of the different cooking method used by those authors. The studies by Yiu et al. consisted of directly adding boiled water to the oats, whereas in our current study the water and oats were mixed together then gradually brought to the boil resulting in differences in the kinetic thermal profile of the oat materials. However, differences in the solid to liquid ratio may also have played a role. We previously hypothesised that starch gelatinisation may explain this phenomenon because the starch will compete with the water-soluble, cell wall polysaccharides for the available free water (Grundy et al., 2017). However, the presence of α-amylase that would remove starch did not result in enhanced release as shown in Fig. 2b.

Cooking appears to be a critical step for the bioaccessibility of oat constituents such as β-glucan. Several mechanisms may be responsible for the low solubilisation of the polymer, including alteration in the cell wall structure and properties (e.g., swelling), lipid coalescence and protein aggregation (Yiu, 1986). Aggregation of lipids and proteins has been shown to reduce their digestibility (Carbonaro et al., 1997, Golding and Wooster, 2010). Thermally induced aggregation of molecules in the matrix could generate a physical barrier/network that would prevent β-glucan from accessing the water and thereby limit its solubilisation (Ulmius et al., 2011).

Regardless of the material and processing method, the weight-average MW of the β-glucan in flour and flakes, measured by the calcofluor method after simulated gastric or duodenal digestion, was ∼1100 × 10^3^ g/mol which was unchanged from the pre-digestion value (*data not shown*). Therefore, digestion or hydrothermal cooking did not influence the molecular size of the β-glucan that was released into solution.

### 3.2 Effect of oat processing on macronutrients digestibility (Digestion 1)

**Fig. 3:**
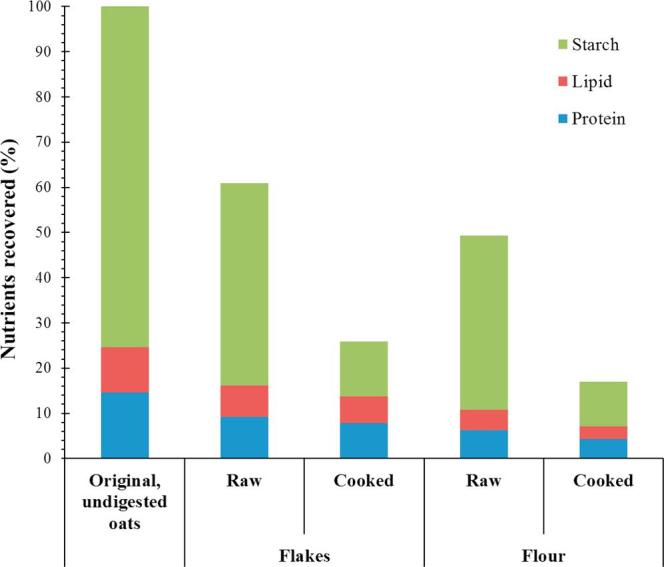
Amount of macronutrients recovered from simulated digestion (oral, gastric and duodenal) of raw and cooked, oat flakes and flour. The data are expressed as a percentage of the total amount of macronutrients present in the original, undigested material (n = 2).

Macronutrient digestibility, as measured by the proportion of macronutrients remaining after digestion, was more extensive for the raw flour than for the raw flakes (Fig. 3). Thus, ∼44% of the total amount of protein and lipid, and 51% of the total starch in raw flour remained undigested compared with higher values of ∼66% of protein and lipid, and 60% of starch observed for digested raw flakes. Hydrothermal processing marginally increased the amounts of lipid and protein that were digested (on average ∼29 and 57% of these nutrients in cooked flour and flakes remained undigested, respectively). However, only a much smaller proportion of the starch was undigested following cooking so that 13 and 16% starch in the cooked flour and flakes, respectively, was recovered after the 4 h simulated digestion. These data demonstrate the importance of processing and oat structures on nutrient digestibility, i.e., different parts of the oat kernel behave differently in accordance with their morphological characteristics (endosperm vs bran). Oat kernels have a heterogeneous macronutrient distribution: the aleurone and subaleurone layers are rich in protein and lipid, whereas the endosperm is comprised primarily of starch (Miller & Fulcher, 2011). Although the protein and lipid are mainly located within the aleurone and subaleurone cells that are more resistant to digestion, some are also found in the endosperm (which represents the majority of the oat kernel). This would account for the increased protein and lipid digestion observed, particularly in the oat flour.

Oat flakes were made by steel cutting, steam tempering and flaking (rolling) whole kilned oat groats. All components of the oat, the bran layers (including the aleurone and subaleurone layers), endosperm and germ were present. Additionally, as the flour were compositionally identical, the only distinction between the two materials was the size of the particles and thus the proportion of fractured cells (Grundy et al., 2017). Consequently, the digestibility of the starch appeared to be essentially similar between flakes and flour. This is presumably because most of the cell walls in the endosperm, which are relatively thinner than the cell walls in the bran layers, were ruptured (as is the case with the oat flour) and/or more porous to digestive enzymes such as amylase, as recently observed in wheat endosperm (Edwards et al., 2014, Edwards et al., 2015a, Edwards et al., 2015b). It is also likely that the starch in the hydrothermally-treated oat flour endosperm, the cells of which are severely ruptured, is gelatinised and therefore more susceptible to amylolysis. Also, in the oat flake samples, the surface of the endosperm will contain ruptured cells with exposed starch granules (raw and gelatinised), which are available for digestion (see microscopy discussion below). Recent studies of wheat endosperm macroparticles have shown that even starch in intact cells are susceptible to the action of α-amylase, possibly due to increased porosity of the cell walls (Edwards et al., 2014, Edwards et al., 2015a, Edwards et al., 2015b). In contrast, the aleurone and subaleurone cells have relatively thicker cell walls, rich in non-starch polysaccharides (notably β-glucan and arabinoxylan) that are highly resistant to digestion in the upper gastrointestinal tract, and such cell walls are likely to remain intact and their contents less accessible to digestive enzymes (Yiu, 1986, Yiu and Mongeau, 1987). The low bioaccessibility of nutrients such as iron in aleurone cells has already been demonstrated in wheat (Latunde-Dada et al., 2014).

**Fig. 4:**
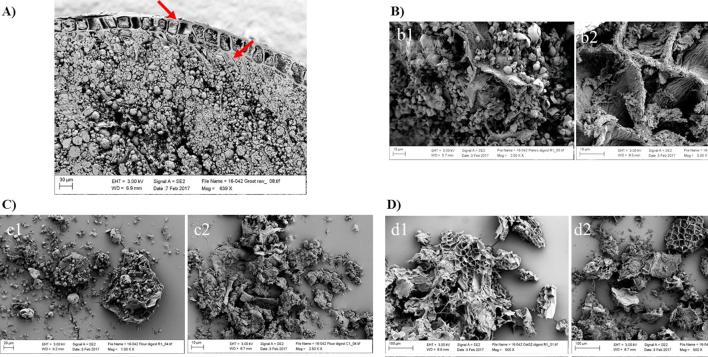
Scanning electron microscopy images of an oat groat section at baseline (not digested) (A), and raw (1) and cooked (2) oat flakes (B), flour (C) and BG32 (D) following 4 h of gastrointestinal digestion. Note the presence of raw starch granules (b1 and c1) and intact cell walls (B, C and D). Note the thick cell walls of the aleurone and subaleurone layers in image A (red arrows). (For interpretation of the references to colour in this figure legend, the reader is referred to the web version of this article.)

The microstructural characteristics of the groat and digested oat materials can be seen in Fig. 4. For both raw and hydrothermally cooked samples, relatively large particles were recovered post-digestion, irrespective of the material. The morphological features of the bran and endosperm in the oat groat can be clearly seen in Fig. 4A which shows the thick aleurone and subaleurone cell walls and the starch granules located in the other parts of the outer endosperm.

The presence of intact cell walls, most likely from the bran, can be observed in all recovered oat particles which is consistent with the β-glucan release data (Fig. 2). Cooking did not seem to enhance the release of this polymer from the oat matrix compared with the raw samples (Fig. 2), which is compatible with the amounts of intact cell wall observed in the digested samples (Fig. 4b2, c2 and d2). Other cell wall components and intra-cellular oat constituents present near the β-glucan may have “entrapped” it and hindered its release and solubilisation (Fig. S1 b3, b4, c3 and c4 of the supplementary material).

There is evidence of non-gelatinised native starch on the fractured surface of raw oat materials (Fig. 4b1 and c1), whereas a fibrous network, most likely formed by gelatinised starch and/or denatured proteins, is apparent at the surface of hydrothermally cooked oat particles, seemingly in close contact with the cell walls (Fig. 4b2 and S1b3, b4, c3 and d4). This phenomenon could potentially have prevented β-glucan release. The content of the cells located at the fractured surfaces of the cooked particles appeared to have been mostly digested, especially for BG32 (Fig. 4d2). In contrast, it is plausible, as illustrated in Fig. 3, that the cells situated underneath these fractured surfaces remain intact and full of undigested nutrients (i.e., lipids and proteins in the aleurone and subaleurone layers), whereas, as discussed above, the encapsulated starch in intact endosperm cells was susceptible to digestion.

### 3.3 Effect of oat processing on lipid digestion

#### 3.3.1 Digestion of intrinsic (intra-cellular) lipids in oat tissue (Digestion 2)

**Fig. 5:**
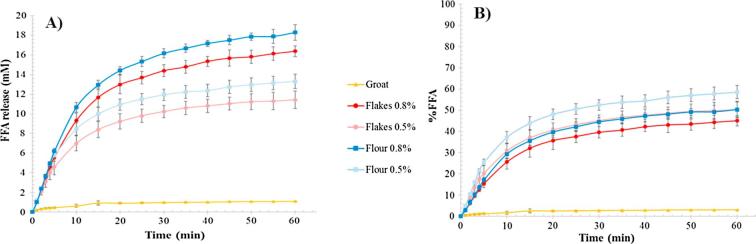
Free fatty acids (FFA) released during the simulated duodenal digestion of raw oat groat (yellow lines), flakes (red lines) and flour (blue lines) at concentrations corresponding to β-glucan contents of either 0.5 or 0.8%. The data are presented as absolute concentrations in mM (A) or as a percentage of the total amount of lipids (triacylglycerols) present in the original material (B) (n = 3). (For interpretation of the references to colour in this figure legend, the reader is referred to the web version of this article.)

The rate and extent of *in vitro* lipolysis monitored using the pH-stat method showed an increase in the amount of FFA generated with respect to the degree of processing (Fig. 5A). Two concentrations of flour or flakes were used, corresponding to a total β-glucan concentration of either 0.5 or 0.8%, and compared with the original groat. As expected, only a small proportion of the lipid contained in the oat groats was digested, which corresponded to only 3% of the lipid originally present in the grain (Fig. 5B). These digested lipids that might have been contained in cells located at the surface of the groats and may have been fractured during the de-hulling process. Similar to the results from Digestion 1, less FFA were generated during the digestion of the oat flakes at both β-glucan concentrations compared with the oat flour. The FFA production for the flakes after 60 min of digestion in the pH-stat system was 11.4 and 16.4 mM for 0.5 and 0.8% β-glucan, respectively, compared with 13.3 and 18.3 mM for 0.5 and 0.8%, β-glucan respectively, for the flour over the same time period. These results suggest that there is a greater degree of lipid encapsulation by the cell walls in the flake sample, which has more intact cells. This encapsulation mechanism of restricting lipid bioaccessibility and digestion has been described in recent studies on almonds (Grundy et al., 2015).

The results were then normalised as a proportion of the total amount of lipids present in the undigested oat material (baseline) (Fig. 5B). The flake and flour oat samples with the highest concentration of starting material (0.8% β-glucan) demonstrated a greater reduction in FFA release during lipolysis than the corresponding samples of lower β-glucan concentration (0.5%). It is worth noting that for the 0.5% β-glucan flake and flour samples, the concentration of β-glucan released into solution after 1 h of digestion was only ∼0.04 and 0.11%, respectively, whereas for the 0.8% β-glucan flake and flour samples, it was ∼0.09 and 0.19%, respectively (*full data not shown*). Therefore, there may be, for a defined MW (here 1100 × 10^3^ g/mol), a range of solubilised β-glucan concentrations that are more effective at influencing lipolysis (see Section 3.3.2). Further studies will need to define this range.

#### 3.3.2 Digestion of extrinsic lipids in presence of pure polysaccharides and oat tissue materials (Digestion 3)

**Fig. 6:**
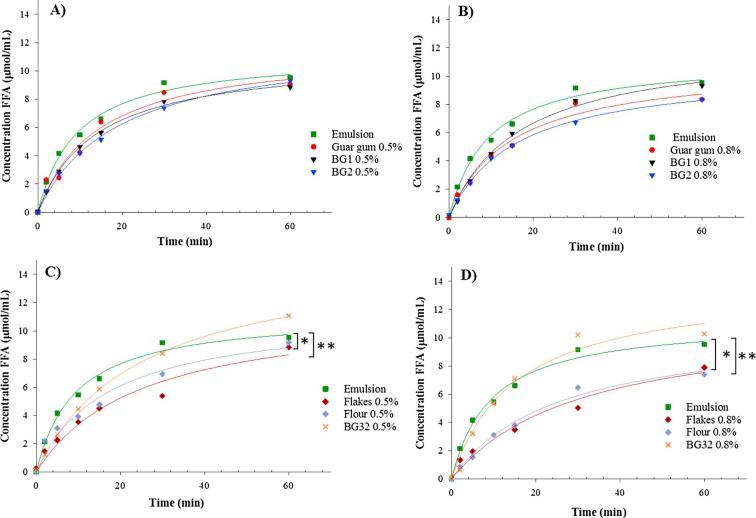
FFA released (μmol/mL) over a 60 min time period during duodenal digestion of sunflower emulsion in the presence of either guar gum, BG1, BG2, oat flakes, oat flour, or oat bran BG32 containing 0.5% (A and C) or 0.8% (B and D) of β-glucan. The data were fitted by non-linear regression using Sigmaplot. Statistical significance was determined using a one-way ANOVA (^*^P < 0.05 and ^**^P < 0.01, flour or flakes vs emulsion, n = 2).

**Fig. 7:**
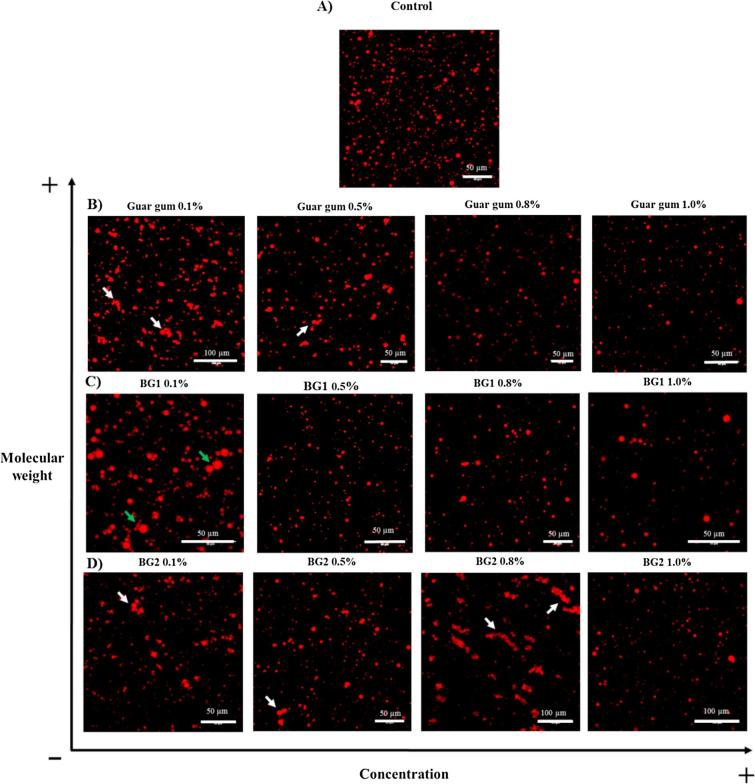
Confocal images of emulsion (A) and emulsion/polymer mixtures at baseline (B to D). Lipids were stained with Nile red. Note the presence of coalesced (green arrow) and depletion-flocculated droplets (white arrow). (For interpretation of the references to colour in this figure legend, the reader is referred to the web version of this article.)

The addition of pure polymers to sunflower emulsions did not have a statistically significant impact on the rate and extent of lipid digestion, and this was a consistent finding regardless of the polymer concentration (Fig. 6A and B). The lack of a clear reduction in FFA release in the presence of these high MW polymers seems, at first sight, to be in disagreement with the literature. Recently, guar gum has been shown to decrease lipolysis by, according to the authors, increasing the digesta viscosity (Amyoony, Lin, & Wright, 2017). However, the guar gum samples used in that study produced a relatively low viscosity when measured in simulated duodenal digesta. Thus, an apparent viscosity of 167 mPa·s at a shear rate of 47.6 s^−1^ was reported for the digesta containing 0.85% guar gum, which suggests that the guar galactomannan was not optimally solubilised and/or it was a low MW grade, unlike that used in the current study. Also, the particle size analysis of the emulsion droplets suggested that there had been significant flocculation or aggregation in the presence of guar galactomannan, which would indicate a reduction in the surface area of lipid available for lipolysis. Another recent study also found that β-glucans of relatively low MW (245 × 10^3^ g/mol) were able to significantly reduce the rate and extent of lipolysis using a synthetic lipid, although the relationship between lipolysis rates and the viscosity of the polymer solution was not uniform (Zhai et al., 2016). The apparent discrepancy with our current results may enable us to shed some light on the mechanisms responsible for these observed decreases in lipid digestion in the literature. Confocal images, shown in Fig. 7, reveal the occurrence of flocculation and/or coagulation of the emulsion droplets, a process that seems dependent on both the size of the polysaccharide and its concentration. It appears that the lower MW (BG2, Fig. 7D) and lower concentrations of both β-glucans and guar galactomannan induced some flocculation and/or coalescence of the emulsion droplets (Fig. 7B and C). Two different processes are likely to take place when emulsion droplets are in the presence of water-soluble polysaccharides such as guar galactomannan or β-glucan. These types of soluble dietary fibres exist in solution as fluctuating ‘random coils’ of glycan chains (Morris, 1992). As the concentration is increased, the polymer coils overlap at a specific, MW-dependent concentration, called the critical concentration (c^∗^) and MW (M_c_), above which entanglement occurs and a visco-elastic network is formed. In the dilute regime, solutions of polymers like β-glucan, which are below c^∗^, are free to move independently through the solvent with little or no interpenetration occurring. Any lipid droplets of a stabilised emulsion are also free to diffuse through the solution. In this case, the polymers can influence the colloidal interactions between the emulsion droplets leading to electrostatic or depletion-driven flocculation (Jenkins & Snowden, 1996). On the other hand, at higher polymer concentrations, above c∗, where polymer chains overlap or interpenetrate, the polymer entanglements will restrict the motion of the emulsion droplets, thus limiting the extent of flocculation (Doublier and Wood, 1995, Ren et al., 2003).

The behaviour of lipid droplets within a polymer solution will not behave in a linear way with respect to concentration, and thus lipid digestion will not be simply dependent on viscosity (Moschakis et al., 2014, Moschakis et al., 2005). This behaviour appears to be consistent with the results of our current study, where the emulsions became less stable (i.e., evidence of flocculation or coalescence) at low concentrations (<0.5%), apart from BG2 (the polymer with the lowest MW) that showed some flocculation at 0.8% (Fig. 7). In fact, an apparent effect of BG2 on lipid digestion (although not statistically significant) was only detected for this particular pure source of oat β-glucan (Fig. 6B). Therefore, for a specific size of a random coil polymer, there is likely to be a concentration range which induces flocculation or coagulation that will reduce the surface area of the lipid phase and hence susceptibility to lipolysis.

**Fig. 8:**
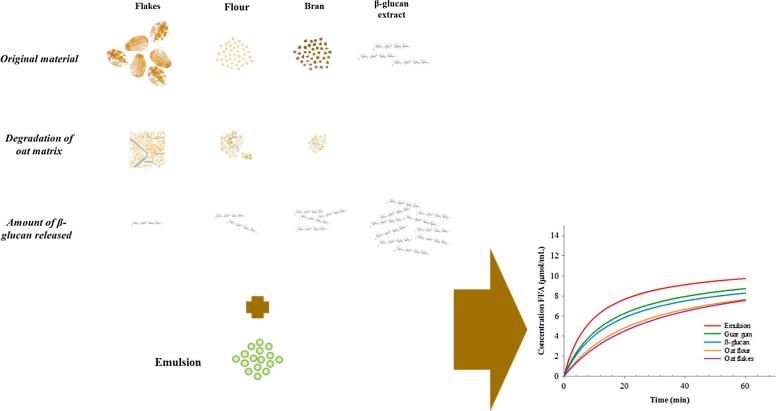
Schematic diagram of the potential mechanisms taking place during lipolysis of sunflower emulsion when in the presence of oat materials (sources of β-glucan) varying in degree of processing. The amount of β-glucan solubilised during digestion is dependent on the composition, structural characteristics, and degree of processing of the oats. *In vitro* digestion experiments showed that the retention of the structural integrity of the oat tissue matrix, and not just the release of β-glucan, is important in hindering lipid digestion.

Lipid digestion experiments performed with oat flour and flakes showed the largest reduction in FFA release (Fig. 6C and D), particularly at the highest polymer concentration; e.g., for the 0.8% β-glucan, there was a reduction in FFA release after 60 min of digestion of 22.3 and 17.0% for flour and flakes, respectively. When comparing the two oat tissue matrices, which vary in particle size only, the absolute amount of β-glucan did not appear to influence lipid digestion greatly. Indeed, as indicated above, the quantity of β-glucan present in solution was lower for oat flakes and flour compared with BG32 and the pure polymer; nevertheless, the oat flake and flour matrices showed the greatest effect in attenuating the rate and extent of lipolysis over 60 min. There are a number of possible mechanisms that may explain the results obtained with the flakes and flour. First, the overall structure of the oat tissue and the specific interaction of the cell walls with the aqueous phase of the digesta may have played a role in its functionality. For example, the β-glucan that has leached from the cell walls may surround the periphery of the oat particles, producing a high concentration of semi-hydrated polymer ('gel-type' layer), which may then potentially interact with digestive agents, especially lipase and co-lipase (Fig. S1 b2 of the supplementary material). Second, components, other than β-glucan, present in the oats, such as starch, proteins, galactolipids, and phytochemicals (e.g., phenolic compounds and phytosterols) may have interfered, alone or in combination, with lipolysis. All these oat compounds, together with the β-glucan, may contribute to the hindrance of lipid digestion at different stages of the digestion process, for instance, during emulsification, micelle formation, removal of the lipid products from the interface, and by inhibition of lipase (Cai et al., 2012, Chu et al., 2009, Jiang et al., 2015). Fig. 8 presents a summary of some of the potential mechanisms that may explain the impact of oats on lipolysis.

Unfortunately, the dynamic aspect of the dissolution of soluble polysaccharides *in vivo* is not possible to replicate currently. When using purified sources, to prevent polymer aggregation, the galactomannan and β-glucan had to be fully dispersed and the mixture of emulsion/polymer made homogeneous before performing the experiments. This solubilisation step may have affected for example the functionality of large MW β-glucan, in particular during the emulsification of the lipid. It is therefore plausible that, in order to have an impact on lipid digestion and cholesterol metabolism, the heterogeneity of the food matrix rich in β-glucan needs to be retained, together with the gradual hydration of the polymer, during, at least, part of the digestion process.

## 4 Conclusions

The current work has used a range of biochemical and biophysical methods, at both the macro- and micro-scales, to improve our understanding of the complex mechanisms occurring during the digestion of lipids in the presence of complex dietary fibre sources. The amount of β-glucan released was dependent on the oat matrix, and a higher level of structural disintegration (flakes vs flour) resulted in an elevated β-glucan solubilisation, whereas hydrothermal treatment decreased the polymer dissolution. The simulated digestion process appeared to have little or no effect on the solubilisation of the β-glucan in any of the oat samples.

However, the time course of lipolysis of the extrinsic lipid using our present emulsion model system did not appear to be directly dependant on the concentration of β-glucan that is released into solution. We have demonstrated that the retention of the structural integrity of the oat tissue matrix is important in reducing the rate and extent of lipolysis. Thus, it is not only the intrinsic functionality of the β-glucan (or other components) that is important, but the physical form by which it is delivered for digestion. Overall, these *in vitro* findings provide some mechanistic insight and explanation of why clinical trials with different oat products (with different degrees of structural integrity of the oat matrix) show varying results on the reduction of blood total and LDL-cholesterol concentrations (Othman et al., 2011, Whitehead et al., 2014).

Further work should include an investigation of the depletion flocculation mechanisms using a wide range of pure soluble polysaccharides varying in MW and concentration. Moreover, it would be valuable to examine the effect of these polymers during the emulsification process. Finally, further studies should investigate the size and composition of micelles generated during digestion, as well as how the lipolysis products and degraded oat material interact with cholesterol and bile and affect their uptake by the enterocytes.
