# Supplementary material for: The impact of oat structure and β-glucan on in vitro lipid digestion
Source: J Funct Foods. 2017 Nov;38(Pt A):378–88. doi: 10.1016/j.jff.2017.09.011 (PMC5666125; doi:10.1016/j.jff.2017.09.011)
Supplement: Supplementary data 1 [file mmc1.docx]

**Appendix A. Supplementary Data**


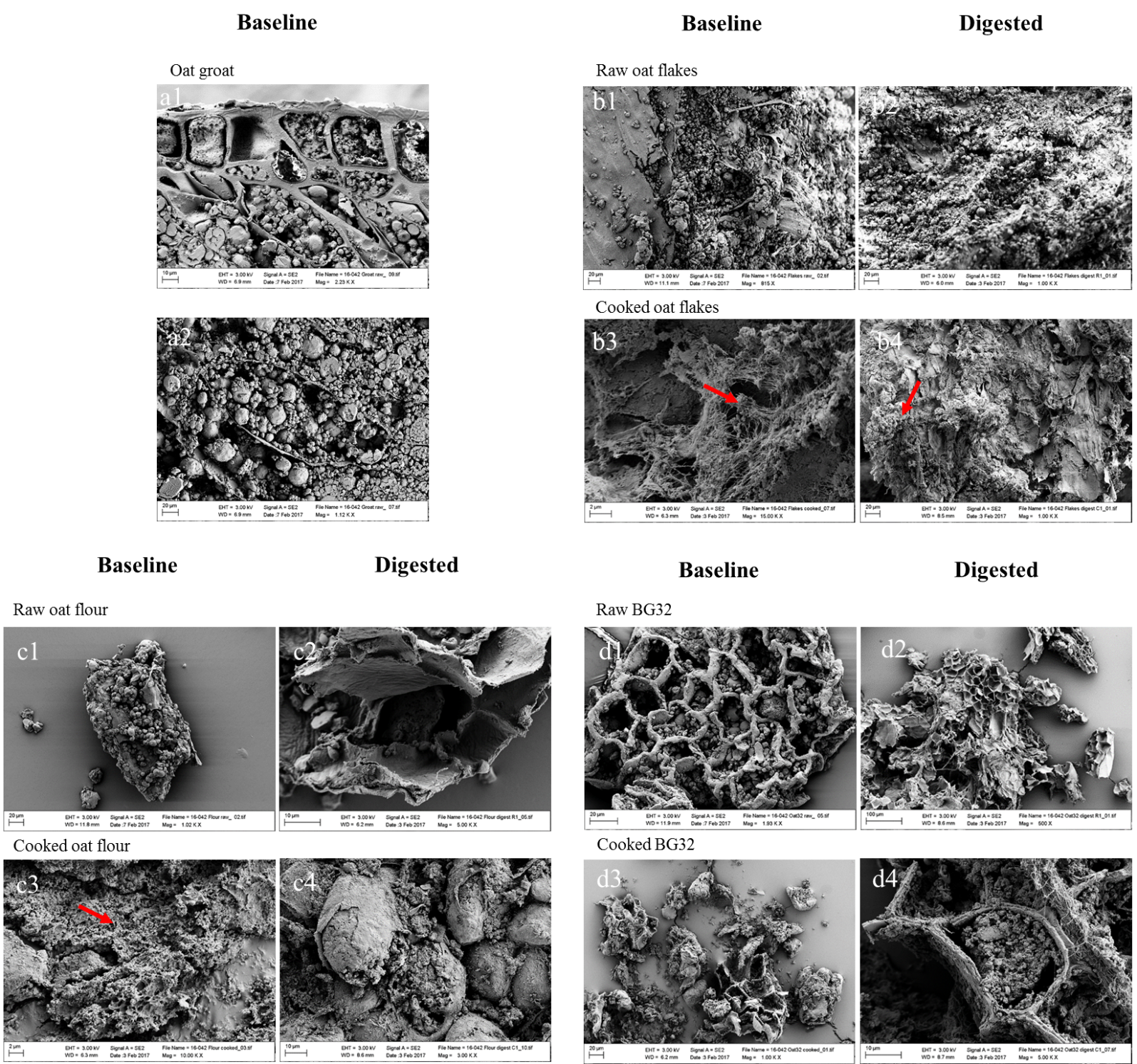


**Fig. S1.** SEM images of raw (1 and 2) and cooked (3 and 4) oat groat (A), oat flakes (B), oat flour (C) and BG32 (D) at baseline (1 and 3) and after 4 h of gastrointestinal digestion (2 and 4). Note in images b3, b4, c3 and c4, the fibrous, gelatinous network in the cooked flakes and flour (red arrows).
